# Supplementary material for: Dual role of the S5 segment in type 1 ryanodine receptor channel gating
Source: Commun Biol. 2024 Sep 18;7:1108. doi: 10.1038/s42003-024-06787-1 (PMC11411075; doi:10.1038/s42003-024-06787-1)
Supplement: Supplementary file 2 — Supplementary Information [file 42003_2024_6787_MOESM2_ESM.pdf]

Supplementary Information for

Dual role of the S5 segment in type 1 ryanodine receptor channel gating

Takashi Murayama[1*], Yuya Otori[2], Nagomi Kurebayashi[1], Toshiko Yamazawa[3], Hideto Oyamada[4], Takashi Sakurai[1], and Haruo Ogawa[2*]

[1]Department of Cellular and Molecular Pharmacology, Juntendo University Graduate School of Medicine, Tokyo 113-8421, Japan

[2]Department of Structural Biology, Graduate School of Pharmaceutical Sciences, Kyoto University, Japan

[3]Core Research Facilities, The Jikei University School of Medicine, Tokyo 105-8461, Japan

[4]Pharmacological Research Center, Showa University, Tokyo 142-8555, Japan

*Correspondence: takashim@juntendo.ac.jp (T.M.); haru@pharm.kyoto-u.ac.jp (H.O.)

Items contained in this file:

Supplementary Figure 1-5

Supplementary Table 1-5

Supplementary References

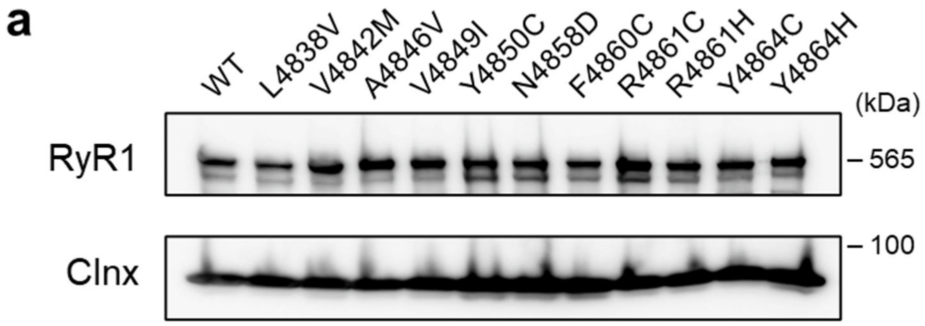

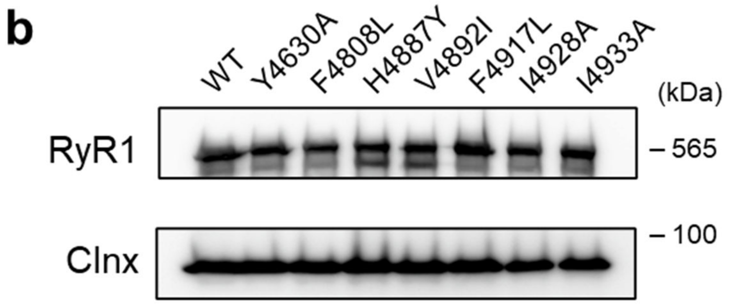

Supplementary Fig. 1: Western blots of RyR1 mutants. a Lysates from HEK293 cells expressing WT and disease-associated RyR1 mutants in the S5 segment were separated by SDS-PAGE and probed with antibodies to RyR (upper panel) and calnexin (Clnx, lower panel) was used as loading control. **b** Similar assay was performed with HEK293 cells expressing RyR1 mutants in the interaction partner of residues for disease-associated mutations.

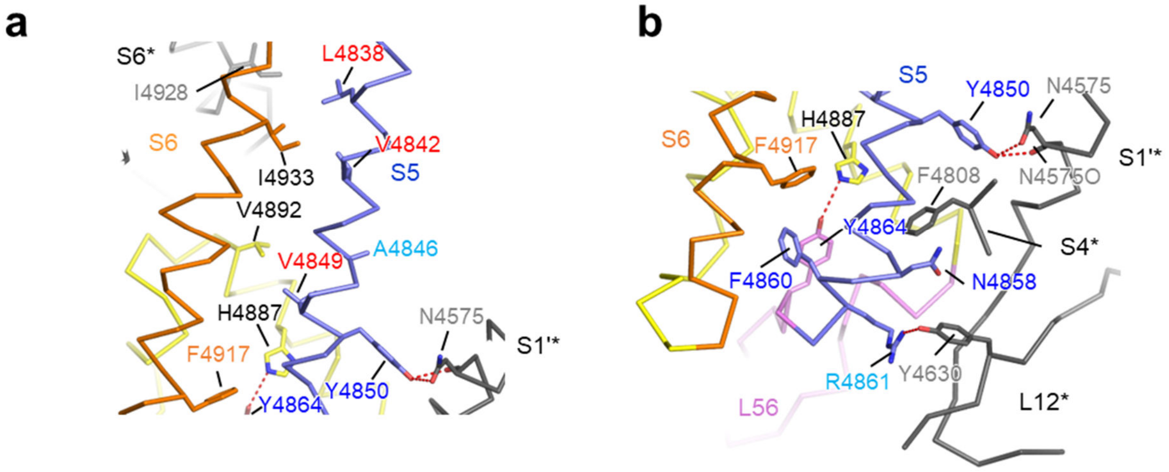

Supplementary Fig. 2: Possible interactions between S5 and the adjacent TM helices based on cryo-EM structures of RyR1 in the open state. **a** Enlarged view of the region in the open state (PDB accession code, 5T15) enclosed by the square dotted line c in Fig.5b. Hydrogen bonds are indicated by red dotted lines. **b** Enlarged view of the region in the open state enclosed by the square dotted line in Fig.5b. Hydrogen bonds are indicated by red dotted lines. S1'*, L12*, S4*, and S6* in gray are the main and side chains from neighboring molecules.

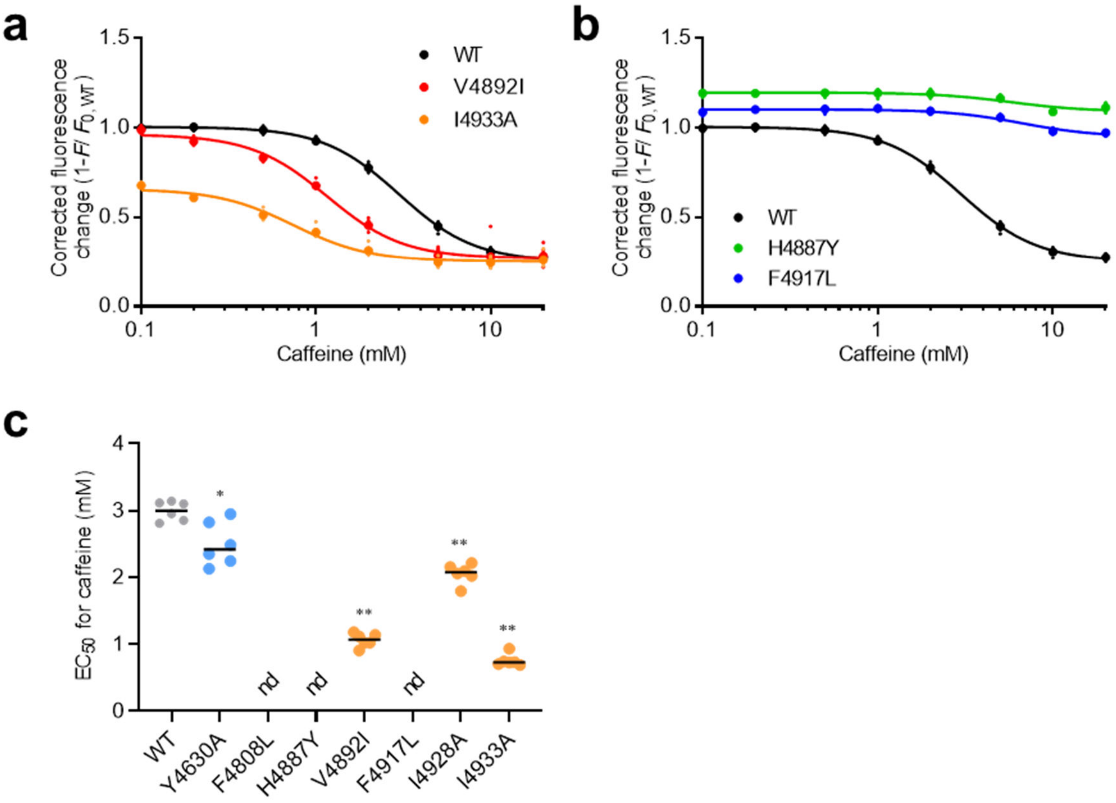

Supplementary Fig. 3: Caffeine-induced Ca^{2+} release activity of the RyR1 channels carrying mutations in the interacting partners. a and **b** Caffeine dependence of the R-CEPIA1er fluorescence corrected by $[Ca^{2+}]_{ER}$ in WT and mutant RyR1s (V4892I and I4933A in **a** and H4887Y and F4917L in **b**). **c** EC_{50} values for caffeine. nd, not determined due to virtually no reduction in $[Ca^{2+}]_{ER}$ by $[K^{+}]$. Data are shown as the means and individual points (n = 6, N = 2). "n" is the number of wells, and "N" is the number of independent experiments.

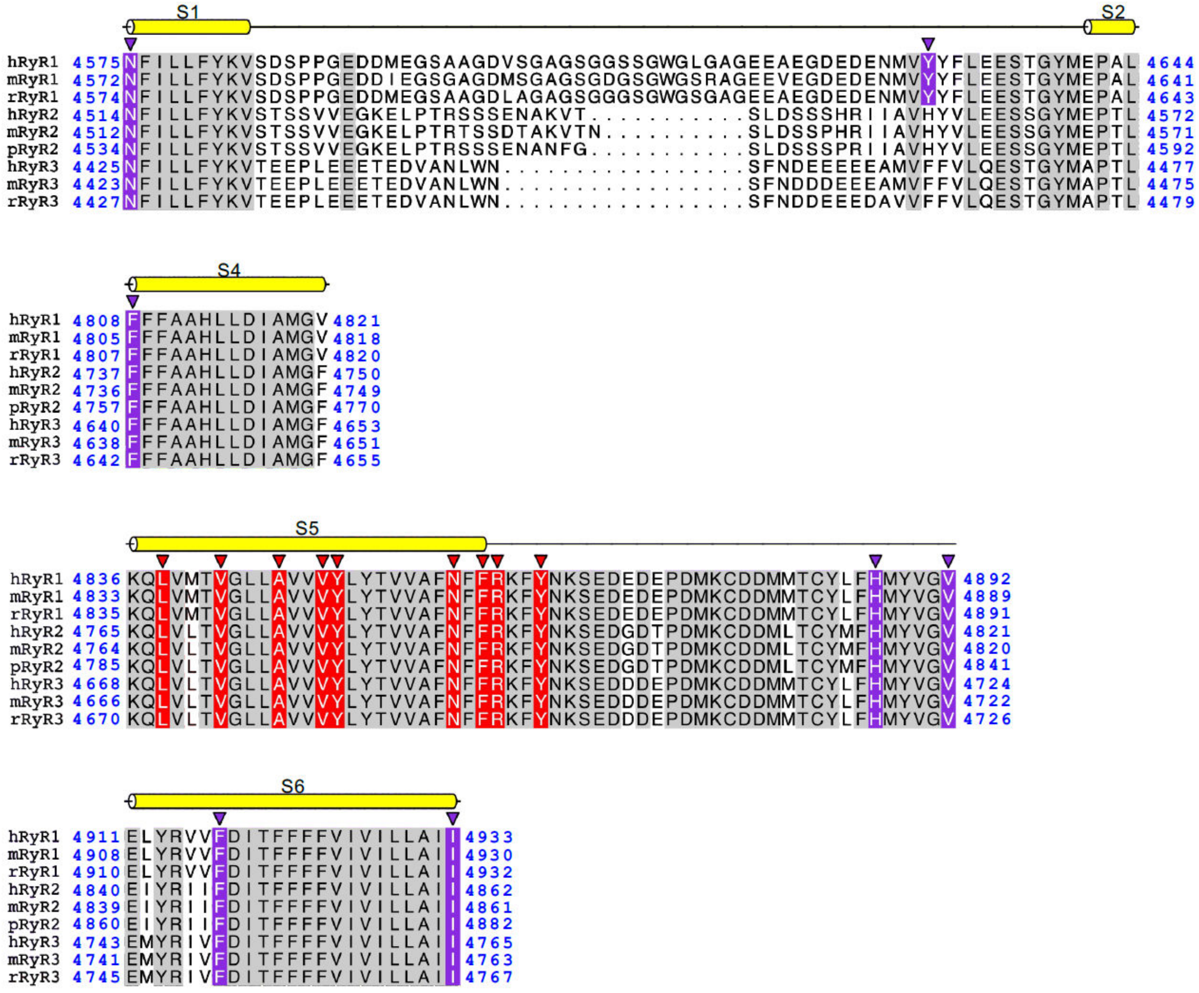

Supplementary Fig. 4: Multiple sequence alignment of three RyR isoforms around disease-associated mutations and the interacting partners. Residues shaded in gray are the identical sequences among isoforms, and residues shaded in red and purple are the sites for the associated mutations in S5 and those for the interacting partners, respectively. Transmembrane segment is shown as a yellow cylinder above the alignment. m, mouse; h, human; p, pig; r, rabbit.

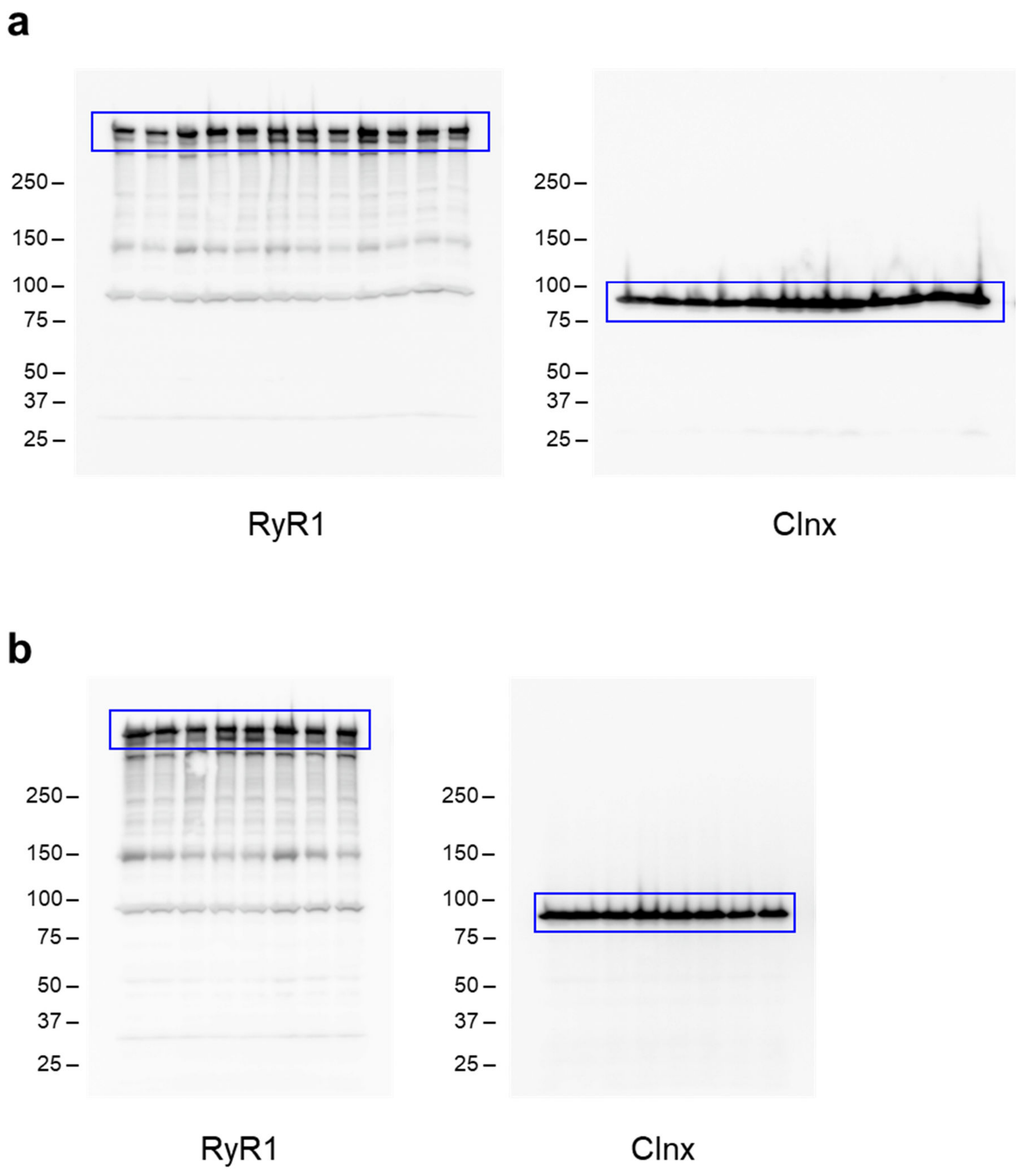

Supplementary Fig. 5: Uncropped blots for Supplementary Fig. 1a (a) and Supplementary Fig. 1b (b). Areas enclosed by blue lines are cropped.

Supplementary Table 1. Disease-associated mutations in S5 and the subsequent beginning of the S5-S6 loop.

Nucleotide change[a]	Protein change[b]	Disease status	Domain[c]	References
c.14512C>G	p.L4838V	MH	S5	[1]
c.14524G>A	p.V4842M	MH	S5	[2]
c.14537C>T	p.A4846V	CCD	S5	[3]
c.14545G>A	p.V4849I	MH	S5	[4]
c.14549A>G	p.Y4850C	CCD	S5	[5]
c.14572A>G	p.N4858D	CCD	S5	[6]
c.14578_14580del	p.F4860del	CCD	S5	[7]
c.14578T>G	p.F4860V	CCD	S5	[8]
c.14581C>T	p.R4861C	CCD	L56	[7, 9]
c.14582G>A	p.R4861H	CCD	L56	[7, 10]
c. 14588_14606del	p.4863-4869delYins	CCD	L56	[11]
c.14591A>G	p.Y4864C	CCD	L56	[12]
c.14590T>C	p.Y4864H	CCD	L56	[13]

[a]Nucleotide number +1 was used as the A of the ATG translation initiation codon in human RyR1 cDNA (GenBank accession number J05200.1).

[b]Residue numbers refer to human RyR1.

[c]Domain is based on the cryo-EM structure of rabbit RyR1. L56 represents the S5-S6 luminal loop.

Supplementary Table 2. Summary of the number of cells (n), the number of dishes (N) and the exact p values compared with WT.

	Ca^{2+} signals by caffeine (Fig. 2c)			Resting $[Ca^{2+}]_{ER}$ (Fig. 2f)			Resting $[Ca^{2+}]_{cyt}$ (Fig. 2g)		
	n	N	p value	n	N	p value	n	N	p value
WT	150	4		88	4		160	3	
L4838V	76	3	<0.0001	68	3	<0.0001	215	3	<0.0001
V4842M	139	4	<0.0001	59	3	<0.0001	157	3	<0.0001
A4846V	70	3	<0.0001	72	3	0.4427	154	3	<0.0001
V4849I	80	3	<0.0001	39	3	<0.0001	196	3	<0.0001
Y4850C	70	3	<0.0001	54	3	0.9951	203	3	0.0895
N4858D	70	3	<0.0001	62	3	0.9202	241	3	0.8731
F4860V	70	3	<0.0001	60	3	0.4988	283	4	<0.0001
R4861C	70	3	<0.0001	62	3	0.7727	321	5	0.6554
R4861H	80	3	>0.9999	75	4	0.1969	109	3	0.0012
Y4864C	70	3	<0.0001	65	3	0.8204	254	3	0.9243
Y4864H	70	3	<0.0001	72	4	0.9177	324	5	<0.0001

Supplementary Table 3. Summary of functional characterization of the disease-associated mutants

Mutants	Caffeine-induced Ca^{2+} release		$[Ca^{2+}]_{cyt}$	$[Ca^{2+}]_{ER}$	$[^3H]$Ry binding	DICR	phenotype
	Max Ca^{2+}	EC_{50}					
L4838V	↓	↓	↑	↓	↑	↑	GOF (severe)
V4842M	↓	↓	↑	↓	↑	↑	GOF (mild)
A4846V	–	↓	–	–	↓	–	LOF (mild)
V4849I	↓	↓	↑	↓	↑	↑	GOF (severe)
Y4850C	↓	nd	–	–	↓	↓	LOF (severe)
N4858D	↓	nd	–	–	↓	↓	LOF (severe)
F4860V	↓	nd	–	–	↓	↓	LOF (severe)
R4861C	–	↑	–	–	↓	↓	LOF (mild)
R4861H	–	↑	–	–	–	–	LOF (mild)
Y4864C	↓	nd	–	–	↓	↓	LOF (severe)
Y4864H	↓	nd	–	–	↓	↓	LOF (severe)

The upward and downward arrows indicate the increases and decreases in the parameters, respectively. The horizontal bars indicate the absence of changes in the parameters. nd, not determined due to no response.

Supplementary Table 4. Possible interacting residues for disease-associated mutations.

Residue	Interacting partner	Site[a]	Disease mutation	Refs	Mutant created	Phenotype
L4838	I4928	S6*	I4928V	[2]	I4928A	GOF
V4842	I4933	S6			I4933A	GOF
V4849	V4892	PH			V4892I	GOF
Y4850	N4575	S1'*			nd[b]	nd[b]
N4858	F4808	S4*	F4808L (CCD)	[14]	F4808L	LOF
F4860	F4917	S6	F4917L (myopathy)	[15]	F4917L	LOF
R4861	Y4630	L12*			Y4630A	No change
Y4864	H4887	PH	H4887Y (myopathy)	[16]	H4887Y	LOF

[a]Asterisks in the site indicate neighboring subunits.

[b]N4575A was not determined (nd) due to difficulty in establishing stable cells.

Supplementary Table 5. List of PCP primers and templates for generation of mutant RyR1.

Mutant	F-primer	R-primer	Template
L4838V	**<u>G</u>**TGGTGATGACTGTGGGCCTC	CTGTTTCCCATTGTGGGTGAC	Cs11
V4842M	**<u>A</u>**TGGGCCTCCTGGCCGTCGTG	AGTCATCACCAGCTGTTTCCC	Cs11
A4846V	**<u>T</u>**CGTCGTGGTCTACCTGTACA	CCAGGAGGCCCACAGTCATCA	Cs11
V4849I	**<u>A</u>**TCTACCTGTACACTGTGGTG	CACGACGGCCAGGAGGCCCAC	Cs11
Y4850C	**<u>G</u>**CCTGTACACTGTGGTGGCCTT	AGACCACGACGGCCAGGAGGC	Cs11
N4858D	**<u>G</u>**ACTTCTTCCGCAAGTTCTAC	GAAGGCCACCACAGTGTACAG	Cs11
F4860V	**<u>G</u>**TCCGCAAGTTCTACAACAAG	GAAGTTGAAGGCCACCACAGT	Cs11
R4861C	**<u>T</u>**GCAAGTTCTACAACAAGAGC	GAAGAAGTTGAAGGCCACCAC	Cs11
R4861H	**<u>A</u>**CAAGTTCTACAACAAGAGCG	GGAAGAAGTTGAAGGCCACCA	Cs11
Y4864C	**<u>G</u>**CAACAAGAGCGAGGACGAGG	AGAACTTGCGGAAGAAGTTGA	Cs11
Y4864H	**<u>C</u>**ACAACAAGAGCGAGGACGAGG	GAACTTGCGGAAGAAGTTGAA	Cs11
Y4630F	**<u>T</u>**CTACTTCCTGGAGGAGAGCA	ATACCATGTTCTCGTCCTCGT	Cs10
F4808L	**<u>C</u>**TCTTCTTTGCCGCCCACCTG	GTTGTTGTAGTGGCCCAGGAG	Cs11
H4887Y	**<u>T</u>**ACATGTACGTGGGCGTCCGG	GAACAGGTAGCACGTCATCAT	Cs11
V4892I	**<u>A</u>**TCCGGGCTGGCGGAGGCATC	GCCCACGTACATGTGGAACAG	Cs11
F4917L	**<u>C</u>**TCGACATCACCTTCTTCTTC	GACCACCCGGTAGAGCTCGTA	Cs11
I4928A	**<u>GC</u>**CCTGCTGGCCATCATCCAGG	GACAATGACGAAGAAGAAGAA	Cs11
I4933A	**<u>GC</u>**CCAGGGTCTGATTATCGACG	GATGGCCAGCAGGATGACAAT	Cs11

Mutated bases are bold and underlined.

Supplementary References

1. Oyamada, H., K. Oguchi, N. Saitoh, T. Yamazawa, K. Hirose, Y. Kawana, K. Wakatsuki, K. Oguchi, M. Tagami, K. Hanaoka, M. Endo, and M. Iino, Novel mutations in C-terminal channel region of the ryanodine receptor in malignant hyperthermia patients. Jpn J Pharmacol, 2002. **88**(2): p. 159-66.
2. Kraeva, N., S. Riazi, J. Loke, W. Frodis, M.L. Crossan, K. Nolan, A. Kraev, and D.H. Maclennan, Ryanodine receptor type 1 gene mutations found in the Canadian malignant hyperthermia population. Can J Anaesth, 2011. **58**(6): p. 504-13.
3. Kossugue, P.M., J.F. Paim, M.M. Navarro, H.C. Silva, R.C. Pavanello, J. Gurgel-Giannetti, M. Zatz, and M. Vainzof, Central core disease due to recessive mutations in RYR1 gene: is it more common than described? Muscle Nerve, 2007. **35**(5): p. 670-4.
4. Carpenter, D., R.L. Robinson, R.J. Quinnell, C. Ringrose, M. Hogg, F. Casson, P. Booms, D.E. Iles, P.J. Halsall, D.S. Steele, M.A. Shaw, and P.M. Hopkins, Genetic variation in RYR1 and malignant hyperthermia phenotypes. Br J Anaesth, 2009. **103**(4): p. 538-48.
5. Bharucha-Goebel, D.X., M. Santi, L. Medne, K. Zukosky, J. Dastgir, P.B. Shieh, T. Winder, G. Tennekoon, R.S. Finkel, J.J. Dowling, N. Monnier, and C.G. Bonnemann, Severe congenital RYR1-associated myopathy: the expanding clinicopathologic and genetic spectrum. Neurology, 2013. **80**(17): p. 1584-9.
6. Wu, S., M.C. Ibarra, M.C. Malicdan, K. Murayama, Y. Ichihara, H. Kikuchi, I. Nonaka, S. Noguchi, Y.K. Hayashi, and I. Nishino, Central core disease is due to RYR1 mutations in more than 90% of patients. Brain, 2006. **129**(Pt 6): p. 1470-80.
7. Monnier, N., N.B. Romero, J. Lerale, P. Landrieu, Y. Nivoche, M. Fardeau, and J. Lunardi, Familial and sporadic forms of central core disease are associated with mutations in the C-terminal domain of the skeletal muscle ryanodine receptor. Hum Mol Genet, 2001. **10**(22): p. 2581-92.
8. Robinson, R., D. Carpenter, M.A. Shaw, J. Halsall, and P. Hopkins, Mutations in RYR1 in malignant hyperthermia and central core disease. Hum Mutat, 2006. **27**(10): p. 977-89.
9. Davis, M.R., E. Haan, H. Jungbluth, C. Sewry, K. North, F. Muntoni, T. Kuntzer, P. Lamont, A. Bankier, P. Tomlinson, A. Sanchez, P. Walsh, L. Nagarajan, C. Oley, A. Colley, A. Gedeon, R. Quinlivan, J. Dixon, D. James, C.R. Muller, and N.G. Laing, Principal mutation hotspot for central core disease and related myopathies in the C-terminal transmembrane region of the RYR1 gene. Neuromuscul Disord, 2003. **13**(2): p. 151-7.
10. Tilgen, N., F. Zorzato, B. Halliger-Keller, F. Muntoni, C. Sewry, L.M. Palmucci, C. Schneider, E. Hauser, F. Lehmann-Horn, C.R. Muller, and S. Treves, Identification of four novel mutations in the C-terminal membrane spanning domain of the ryanodine receptor 1: association with central core disease and alteration of calcium homeostasis. Hum Mol Genet, 2001. **10**(25): p. 2879-87.
11. Zorzato, F., N. Yamaguchi, L. Xu, G. Meissner, C.R. Muller, P. Pouliquin, F. Muntoni, C. Sewry, T. Girard, and S. Treves, Clinical and functional effects of a deletion in a COOH-terminal lumenal loop of the skeletal muscle ryanodine receptor. Hum Mol Genet, 2003. **12**(4): p. 379-88.
12. Quinlivan, R.M., C.R. Muller, M. Davis, N.G. Laing, G.A. Evans, J. Dwyer, J. Dove, A.P. Roberts, and C.A. Sewry, Central core disease: clinical, pathological, and genetic features. Arch Dis Child, 2003. **88**(12): p. 1051-5.
13. Cacheux, M., A. Blum, M. Sebastien, A.S. Wozny, J. Brocard, K. Mamchaoui, V. Mouly, N. Roux-Buisson, J. Rendu, N. Monnier, R. Krivosic, P. Allen, A. Lacour, J. Lunardi, J. Faure, and I. Marty, Functional Characterization of a Central Core Disease RyR1 Mutation (p.Y4864H) Associated with Quantitative Defect in RyR1 Protein. J Neuromuscul Dis, 2015. **2**(4): p. 421-432.
14. Kraeva, N., E. Zvaritch, A.E. Rossi, S.A. Goonasekera, H. Zaid, W. Frodis, A. Kraev, R.T.

Dirksen, D.H. Maclennan, and S. Riazi, Novel excitation-contraction uncoupled RYR1 mutations in patients with central core disease. Neuromuscul Disord, 2013. **23**(2): p. 120-32.

15. Maggi, L., M. Scoto, S. Cirak, S.A. Robb, A. Klein, S. Lillis, T. Cullup, L. Feng, A.Y. Manzur, C.A. Sewry, S. Abbs, H. Jungbluth, and F. Muntoni, Congenital myopathies--clinical features and frequency of individual subtypes diagnosed over a 5-year period in the United Kingdom. Neuromuscul Disord, 2013. **23**(3): p. 195-205.

16. Herasse, M., K. Parain, I. Marty, N. Monnier, A.M. Kaindl, J.P. Leroy, P. Richard, J. Lunardi, N.B. Romero, and A. Ferreiro, Abnormal distribution of calcium-handling proteins: a novel distinctive marker in core myopathies. J Neuropathol Exp Neurol, 2007. **66**(1): p. 57-65.
