# Supplementary material for: Dual role of the S5 segment in type 1 ryanodine receptor channel gating
Source: Commun Biol. 2024 Sep 18;7:1108. doi: 10.1038/s42003-024-06787-1 (PMC11411075; doi:10.1038/s42003-024-06787-1)
Supplement: Supplementary file 3 — Description of additional supplementary files [file 42003_2024_6787_MOESM3_ESM.pdf]

Description of Additional Supplementary Files

File name: Supplementary Movie 1

Description: Movement of RyR1 from the closed state (PDB accession code, 5TB0) to the open state (PDB accession code, 5T15) and location of disease-associated mutations.

File Name: Supplementary Movie 2

Description: Details of amino acid residues that interact with the residues of disease-associated mutations in the closed state (PDB accession code, 5TB0).

File name: Supplementary Data

Description: The source data behind the graphs in the paper
